# A child presenting with acute renal failure secondary to a high dose of indomethacin: a case report

**DOI:** 10.1186/1752-1947-3-47

**Published:** 2009-02-03

**Authors:** Felipe González, Jesús López-Herce, Cinta Moraleda

**Affiliations:** 1 Pediatric Intensive Care Unit, Hospital General Universitario Gregorio Marañón, Universidad Complutense de Madrid, Madrid, Spain

## Abstract

**Introduction:**

Acute renal failure caused by nonsteroidal anti-inflammatory drugs administered at therapeutic doses is generally mild, non-anuric and transitory. There are no publications on indomethacin toxicity secondary to high doses in children. The aim of this article is to describe acute renal failure secondary to a high dose of indomethacin in a child and to review an error in a supervised drug prescription and administration system.

**Case presentation:**

Due to a medication error, a 20-day-old infant in the postoperative period of surgery for Fallot's tetralogy received a dose of 10 mg/kg of indomethacin, 50 to 100 times higher than the therapeutic dose. The child presented with acute, oligo-anuric renal failure requiring treatment with continuous venovenous renal replacement therapy, achieving complete recovery of renal function with no sequelae.

**Conclusion:**

In order to reduce medication errors in critically ill children, it is necessary to develop a supervised drug prescription and administration system, with controls at various levels.

## Introduction

Drug toxicity causes 2% to 5% of hospital admissions [1,2]. In addition, between 7% and 10% of hospitalized patients suffer adverse drug reactions [2]. These reactions may be related to the drug (toxic potential, dose, duration, route of administration and interactions with other drugs) or to the patient (age, sex, metabolic abnormalities or associated pathology that could alter drug metabolism and/or excretion). For these reasons, adverse drug reactions are more common in critically ill patients [3].

Many drugs cause renal toxicity. The lesion most commonly develops in the tubules and interstitium but can also affect the glomerulus or intrarenal blood vessels [4]. The risk of drug-induced renal toxicity is higher in children as the glomerular filtration rate is lower and the kidneys have an immature enzyme system. The accidental, voluntary or iatrogenic administration of drug overdoses is a relatively common cause of acute renal failure (ARF) [5]. In children, although the most common causes of ARF are ischemia after cardiac surgery, sepsis and the hemolytic uremic syndrome, drug toxicity can account for up to 16% of cases [6].

Nonsteroidal anti-inflammatory drugs (NSAIDs), including indomethacin, cause renal toxicity by inhibiting the enzyme cyclooxygenase in the glomerulus, producing vasoconstriction [4,5,7,8]. Acute renal failure caused by the NSAIDs administered at therapeutic doses is an underestimated complication as it is usually mild, transitory and non-anuric. In premature neonates, it has been estimated that therapeutic doses of indomethacin give rise to transitory renal toxicity in 24% of cases [9]. However, in the literature, we have found no case reports of acute renal failure in children secondary to the administration of a high dose of indomethacin.

## Case presentation

The patient was a 20-day-old Spanish male infant with a body weight of 2.5 kg, transferred from the neonatal unit on the ninth day after the surgical correction of Fallot's tetralogy and pulmonary atresia. On admission to the pediatric intensive care unit, the infant required mechanical ventilation, an infusion of vasoactive drugs (dopamine 6 mcg/kg/minute, dobutamine 10 mcg/kg/minute and milrinone 0.5 mcg/kg/minute) and furosemide in a continuous infusion of 0.4 mg/kg/hour. The patient had received treatment with vancomycin and amikacin up to 2 days earlier. On examination, there was marked generalized edema. The initial blood tests revealed a creatinine level of 0.5 mg/dL, urea 75 mg/dL, albumin 2.8 g/dL, sodium 132 mmol/L, potassium 4.6 mmol/L and chloride 96 mmol/L. In order to decrease the doses of intravenous vasoactive drugs, it was decided to administer digitalis to the patient, prescribing a dose of digoxin of 10 mcg/kg enterally. Four hours after the administration of the drug, the child presented with progressive oliguria, with a fall in diuresis from 4 to 1.5 mL/kg/hour, and with no change in the hemodynamic situation (blood pressure 65/40 mmHg, lactate 1.1 mmol/L, heart rate 140 bpm). Blood tests revealed a rise in creatinine to 0.7 mg/dL and in urea to 89 mg/dL and a fall in sodium to 121 mmol/L. There were no neurological clinical symptoms or alterations in the cerebral echography that suggested cerebral edema. The urinalysis was normal. Initially, to exclude hypovolemia, volume expansion was performed with 5% albumin (20 mL/kg). Intravenous sodium replacement was started according to the equation (135 - 121) × 0.6 × weight (kg) in 24 hours and the dose of dopamine was increased from 7.5 to 15 mcg/kg/minute in order to raise the mean blood pressure and improve renal perfusion. Subsequently, on persistence of the oliguria, the infusion of furosemide was increased from 0.4 to 1 mg/kg/hour, though no improvement in the diuresis was achieved. The medication chart was reviewed and the error was detected. Instead of digoxin, indomethacin had been prescribed at a dose of 25 mg (10 mg/kg), which is 50 to 100 times higher than the therapeutic dose.

Initially, it was decided to maintain a conservative approach. The child remained hemodynamically stable but presented with anuria and an increase in edema; continuous venovenous hemofiltration was therefore started after 12 hours. This therapy was continued for 33 hours, with a negative balance of 25 mL/hour, after which diuresis and renal function recovered (Table 1). The subsequent course was favorable and the infant was discharged from the pediatric intensive care unit with a creatinine level of 0.2 mg/dL, urea 15 mg/dL and normal diuresis. No alteration of renal function has been detected on subsequent follow-up.

**Table 1:** Evolution of analytical data

	Admission to the PICU	Start of CRRT	End of CRRT
Na (mmol/L)	132	121	129
K (mmol/L)	4.6	4.1	2.9
Urea (mg/dL)	57	89	34
Creatinine (mg/dL)	0.5	0.7	0.4
Urine output (mL/kg/hour)	4.5	0	3

PICU, pediatric intensive care unit; CRRT, continuous renal replacement therapy.

## Discussion

NSAIDs are a relatively common cause of renal toxicity in the adult [4,6]. These drugs can lead to renal failure through various mechanisms: the most important of these is the decrease in the levels of prostaglandins, which regulate arterial and glomerular vasodilatation, though they can also produce acute tubular necrosis due to direct toxicity or to acute interstitial nephritis [4,5,7,8].

Indomethacin is a drug that is not commonly used in children. However, it is the treatment of choice in patent ductus arteriosus in the premature newborn infant. In these cases, treatment at a dose of 0.1 mg/kg every 24 hours for 6 days has been reported to be associated with an increase in creatinine levels in up to 24% of patients between the second and seventh days of treatment, with complete recovery of renal function at 30 days [9]. We have found no previous cases of the administration of massive doses of indomethacin in children.

There is a higher risk of drug-induced nephrotoxicity in critically ill patients due to the frequent association with other factors such as pre-existing renal failure or renovascular disease, the presence of secondary hypovolemia, hypertension, cardiac failure, hypoalbuminemia, electrolyte disturbances, hepatic disorders affecting metabolism, and the administration of other drugs that interfere with the metabolism and/or potentiate the nephrotoxicity of indomethacin [10]. In our patient, the administration of indomethacin at a dose 100 times higher than the therapeutic dose was associated with several factors that could have increased its toxicity, such as renal immaturity due to age and cardiac failure secondary to surgery for the congenital cardiopathy, in addition to edema, hypoalbuminemia and the administration of high doses of furosemide.

In our patient, the indomethacin-induced nephrotoxicity led to anuria, requiring the early initiation of continuous renal replacement therapy as hypervolemia secondary to anuria could have led to significant hemodynamic decompensation in a patient who had recently undergone cardiac surgery. In the majority of cases of ARF associated with therapeutic doses of NSAIDs published in the literature, renal function recovered completely within a short period of time [4,7]. Recently, it has been suggested that children who develop ARF have a higher morbidity and mortality and more long-term renal sequelae [11]. However, in our patient, despite the severity of the ARF, recovery of renal function was complete within a few days.

Various studies have demonstrated that a significant number of errors in the prescription and/or administration of medication occur in intensive care units due, in part, to the large number of drugs used, the need for rapid action, and an overburdening of resources [12,13]. Although the majority are relatively unimportant, some can put the patient's life at risk [12,13]. The existence of a supervision system is therefore essential. In our hospital, there is a computerized therapeutic prescription program, with a system of dose error alarms. It is routinely the resident who writes the prescription and this is supervised by the staff physician and confirmed by the nurse responsible for the patient. However, in this patient, the erroneous prescription written by the resident was not detected by the Pharmacy's computerized system and was not checked by the staff physician. The nurse requested confirmation of the drug and dose and this was given by the resident. Although computerized prescription systems have been shown to reduce the number of errors [14,15], it is important to monitor drug prescription and administration at various levels in order to prevent errors; this should involve not only computer systems but also control by the pharmacy staff and, principally, by the medical and nursing staff. Furthermore, if possible, the use of nephrotoxic drugs should be avoided, particularly in combination; when their administration is unavoidable, the need for monitoring of blood levels and for periodic controls of renal function to adjust the dose should be evaluated.

## Conclusion

We conclude that indomethacin at very high doses causes transitory acute renal failure. To reduce medication errors in critically ill children, it is necessary to develop a supervised drug prescription and administration system with controls at various levels.

## Abbreviations

ARF: acute renal failure; NSAIDs: non-steroidal anti-inflammatory drugs.

## Competing interests

The authors declare that they have no competing interests.

## Authors' contributions

FG, JL-H, and CM participated in the design, data collection and analysis, and drafting of the manuscript.

## Consent

Written informed consent was obtained from the patient's parents for publication of this case report. A copy of the written consent is available for review by the Editor-in-Chief of this journal.
